# The scanning four-bounce monochromator for beamline I20 at the Diamond Light Source

**DOI:** 10.1107/S1600577518008974

**Published:** 2018-08-02

**Authors:** Shusaku Hayama, Graham Duller, John P. Sutter, Monica Amboage, Roberto Boada, Adam Freeman, Luke Keenan, Brian Nutter, Leo Cahill, Pete Leicester, Ben Kemp, Nico Rubies, Sofia Diaz-Moreno

**Affiliations:** a Diamond Light Source, Didcot, Oxfordshire OX11 0DE, UK

**Keywords:** X-ray absorption spectroscopy, beamline, monochromator, optics

## Abstract

A description of the technical and design details of a scanning four-bounce crystal monochromator that has recently been commissioned for the Versatile X-ray Absorption Spectroscopy (XAS) beamline at Diamond Light Source is presented. This device consists of two independent rotary axes of unique design which are synchronized using a multiple read-head encoder system. This monochromator is shown to be capable of maintaining the flux throughput of the Bragg axes without the need of any external feedback mechanism from 4 to 20 keV. The monochromator is currently equipped with cryogenically cooled crystals with the upstream axis consisting of two independent Si(111) crystals and a pair of channel-cut crystals in the downstream axis. The possibility of installing an additional Si(311) crystal-set to extend the energy range to 34 keV is incorporated into the preliminary design of the device. Experimental data are presented showing the exceptional mechanical stability and repeatability of the monochromator axes.

## Abstract 2

A scanning four-bounce monochromator that offers an unprecedented level of stability in the incident beam energy resolution and calibration has been developed for a flagship spectroscopy beamline at Diamond Light Source.

## 1. Introduction   

Beamline I20 is the Versatile X-ray Absorption Spectroscopy (XAS) beamline at Diamond Light Source. I20 is a double beamline, with two independent branches. The scanning branch of I20 is designed to perform X-ray absorption near-edge structure (XANES), extended X-ray absorption fine structure (EXAFS) and X-ray emission spectroscopy (XES) experiments. Owing to its careful design, this branchline is capable of operating simultaneously and independently from the second, time-resolved energy-dispersive branch line, which began operation in 2015. The separation of the two branches is achieved by the use of two canted wigglers that deliver X-rays to two dedicated experimental hutches, one for each branch line. The two branches share the same optics hutch due to the limited horizontal separation of the X-ray beams produced by the insertion devices, but they become completely independent afterwards (Diaz-Moreno *et al.*, 2009 ▸, 2018 ▸).

**Figure 1:**
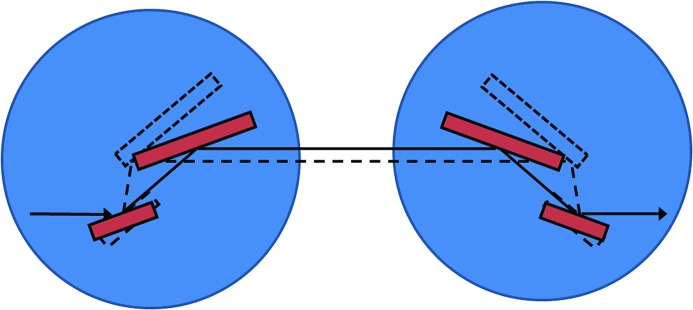
Optical layout of the four-crystal monochromator. The crystals (red) are mounted on two independent (upstream and downstream) axes and rotated in counter directions to select different energies.

The optical configuration of the scanning branch of I20 has been designed to deliver tunable monochromatic X-rays in the range from 4 to 34 keV with high spectral purity and stability without compromising flux throughput. The source of this branch line is a 2 m hybrid wiggler, 83 mm period, which produces a high-intensity X-ray beam with a continuous energy profile. The X-ray beam is conditioned by a series of mirrors housed in the optics hutch, and it is monochromated by the novel four-bounce monochromator, which forms the heart of the scanning branch. The monochromator is formed by four crystals working in a +−−+ configuration that is dispersive between the second and third crystals (see Fig. 1 ▸). Due to the large X-ray source-related heat loads that the first crystal of the monochromator needs to cope with, the crystals are cryogenically cooled. Currently, the monochromator is operating with Si(111) crystals to cover the energy ranges from 4 to 20 keV but there is a plan to extend the operation range to 34 keV using a set of Si(311) crystals once we have addressed the issue of how to effectively cool these crystals.

The concept of an X-ray monochromator using multiple reflections to improve the resolution of the device is not new; it was first proposed by DuMond in 1937 (DuMond, 1937 ▸). Many years later, in 1974, Beamont and Hart, using the concept of the four-crystal reflection in dispersive geometry given by DuMond, made a prototype to be used with synchrotron radiation (Beaumont & Hart, 1974 ▸). Over the years, slightly modified designs based on this principle have been developed, mainly for high-resolution diffraction studies, where there is no need for scanning over a wide range of photon energies (Bartels, 1983 ▸; van der Sluis, 1994 ▸; Loxley *et al.*, 1995 ▸; Servidori, 2002 ▸). For these non-spectroscopic uses, precise synchronization of the Bragg axes is not required. The main technical difficulty that to date has hampered the development of scanning four-bounce monochromators for spectroscopic experiments is the requirement for two highly stable and precisely controllable axes to maintain the Bragg condition between the first and second crystal pairs. Despite this difficulty, a few scanning monochromators have been developed and installed on bending-magnet beamlines around the world [BESSY II (Krumrey, 1998 ▸; Krumrey *et al.*, 1998 ▸; Krumrey & Ulm, 2001 ▸), NSLS (Trela *et al.*, 1988 ▸; Heald, 1984 ▸, 1988 ▸; Heald *et al.*, 1986 ▸; Sayers *et al.*, 1983 ▸), HASYLAB (Kraft *et al.*, 1996 ▸) and LNLS (Tolentino & Rodrigues, 1992 ▸; Tolentino *et al.*, 1995 ▸, 1998 ▸)]. These devices were equipped with water-cooled crystals as the limited power of the bending-magnet sources did not require cryogenic cooling technology. Most recently, a prototype water-cooled four-bounce monochromator built at the SRS Daresbury Laboratory was tested on the test beamline, B16, at Diamond Light Source, demonstrating the technical feasibility of the device. In this paper, we demonstrate that recent advances in motion control technology and drive mechanisms now make possible the construction of a robust four-crystal monochromator that can be routinely operated and scanned across a wide energy range without the need for any external feedback system to maintain the flux throughput of the Bragg axes.

The advantages of four-crystal X-ray monochromators over the conventional double-crystal devices have already been discussed in detail (Servidori, 2002 ▸). For instance, the dispersive nature of the four-crystal monochromator ensures that the energy resolution is unaffected by changes in the divergence of the incoming beam, and make this solely dependent on the intrinsic energy resolution of the crystals (Krumrey, 1998 ▸; Trela *et al.*, 1988 ▸; Heald *et al.*, 1986 ▸; Heald, 1988 ▸; Kraft *et al.*, 1996 ▸; Tolentino & Rodrigues, 1992 ▸). Other benefits include: (i) the fixed exit of the monochromatic beam without the need for translation of any of the crystals (Krumrey, 1998 ▸; Krumrey & Ulm, 2001 ▸; Heald, 1988 ▸; Tolentino & Rodrigues, 1992 ▸); (ii) the variations in the source angular position do not affect the position stability of the exit beam (Tolentino *et al.*, 1995 ▸); and (iii) the intrinsic suppression of the reflectivity curve tails further assists the delivery of high-energy resolution (Krumrey, 1998 ▸; Heald *et al.*, 1986 ▸; Kraft *et al.*, 1996 ▸; Tolentino & Rodrigues, 1992 ▸). These characteristics make the four-crystal monochromator ideal for spectroscopy beamlines such as I20, or any other beamlines with key criteria of high spectral purity and beam stability at the sample position.

In this paper we present a full technical description of the four-bounce monochromator that has been designed and built for beamline I20 at Diamond Light Source and show how the performance of the Bragg axes was tested and successfully characterized. X-ray measurements acquired in transmission and fluorescence detection mode using this monochromator are also presented and discussed. The theoretical considerations that were critical to the design and feasibility of the monochromator have been discussed in a previous publication (Sutter *et al.*, 2008 ▸), and here we restrict our attention to the practical implementation of the device.

## 2. Technical description   

The I20 four-bounce monochromator has been designed, assembled and commissioned in-house at Diamond Light Source. The design of the monochromator is essentially based on four main components: (i) two independent high-precision rotary Bragg axes; (ii) two different cryogenically cooled crystal cages for the two different axes; (iii) a large granite base with a linear air-bearing stage to allow for selection of different crystal sets for future upgrade; and (iv) a central vessel carrying a diagnostic stick. The Bragg axes are mounted on a single heavy-duty frame and the centres of rotation are separated by a distance of 1000 mm. The upstream axis is securely fitted whilst the downstream axis is mounted kinematically to this frame. The frame rests in the large granite base in order to minimize vibrations transmitted from the experimental hall floor. The entire monochromator axes can be translated perpendicular with respect to the beam by using the linear air-bearing located in the granite base, avoiding the need for in-vacuum translations.

The rotary axes have been specifically designed to meet the high repeatability, accuracy and stability demands of the four-crystal monochromator. For this type of monochromator to work, the two Bragg axes need to be synchronized to keep the Bragg condition as the energy is scanned. For beamline I20 this requirement is most stringent when operating at high energies where the Darwin width is narrowest. Our earlier work has shown that an angular resolution of the order of ±0.3 µrad is required to allow the monochromator to work optimally at the highest possible energy of 34 keV using the Si(311) crystals (Sutter *et al.*, 2008 ▸).

**Figure 2:**
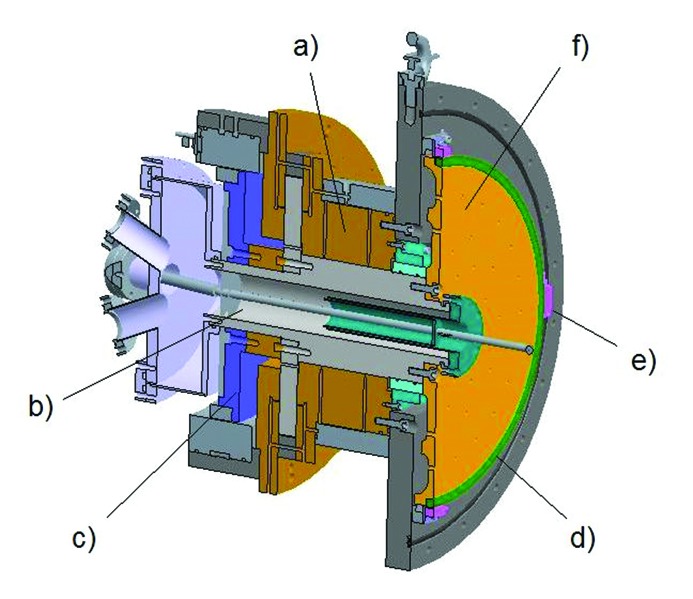
Schematic of the rotary axis showing the main components: (*a*) air bearing; (*b*) borehole used to pass the liquid-nitrogen and water lines to the vacuum side of the monochromator; (*c*) direct-drive motor; (*d*) encoder ring; (*e*) encoder read-heads and (*f*) bronze disk.

The two axes are identical in construction and can be operated independently or simultaneously. A schematic design of the rotator axis is shown in Fig. 2 ▸. At the centre of the axis is a shaft which is supported by two large air bearings (Fluid Film Devices, Romsey, UK) that are operated using 5.1 bar of compressed air. These bearings give high radial and longitudinal stiffness with no mechanical contact between the shaft and the bearings. The use of the air bearings also ensures that the radial run-out of the shaft is kept to sub-micrometre levels. At the rear end of the shaft is a large-diameter direct-drive motor with 66 magnetic poles (ETEL SA, Switzerland). This direct-drive system has been chosen as it offers backlash-free positioning, high dynamic stability and precise feedback control. The shaft and the air bearings are isolated from the vacuum side of the monochromator using a Ferrofluidic^®^ seal (Ferrotec, USA). The seal is designed to withstand greater than 1 bar differential pressure and allows the monochromator to operate at the nominal pressure of 10^−8^ mbar. The presence of the seal adds a considerable amount of viscous drag to the system but this still allows the shaft to be rotated smoothly without any mechanical linkage to the rotation axis.

**Figure 3:**
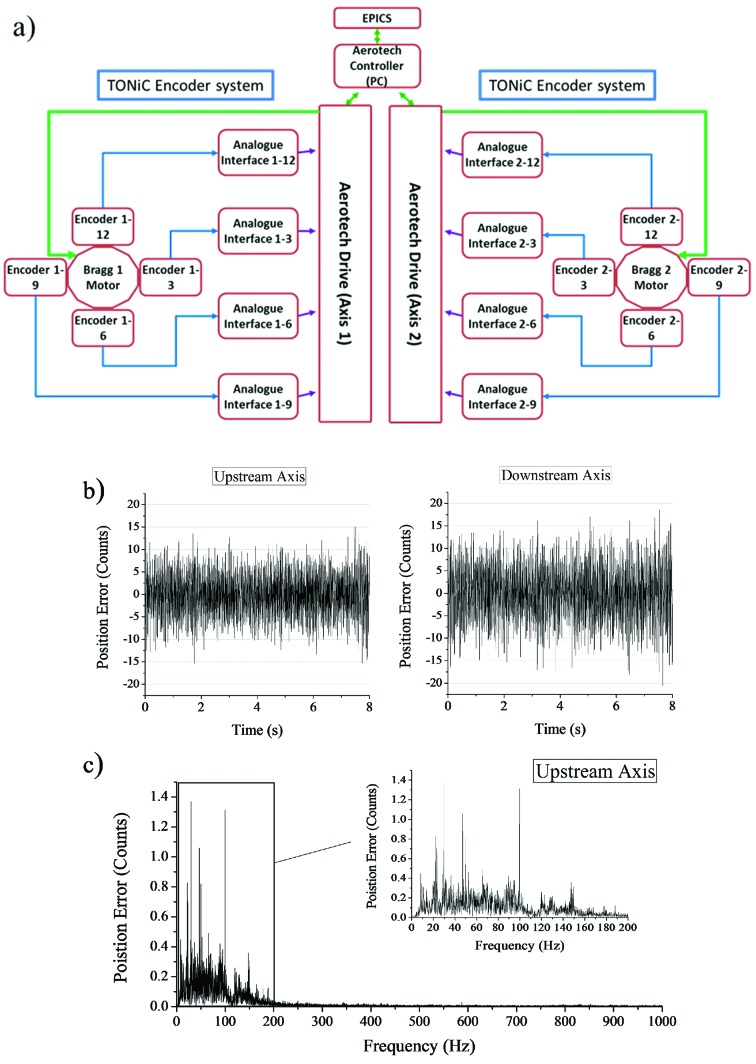
(*a*) Schematic layout of the control system with two independent drive systems for the upstream and downstream axes. (*b*) Typical positional errors reported by the control system when the monochromator is under real operating conditions with the crycooler liquid-nitrogen pump speed set at 30 Hz. (*c*) Fourier transform of (*b*). The peak-to-peak error reported is consistently worse for the downstream axis and it slightly exceeds 150 nrad but the majority of steps are within ±15 counts (30 counts × 4.848 nrad = 145 nrad).

The direct-drive motors are controlled using the positional feedback from encoders and using a drive controller system. The drive system needed a significant amount of attention and had to go through several iterations before a practical solution was found that could meet all the necessary requirements. The key difficulty here was ensuring that the overall jitter level did not exceed ±0.075 µrad and at the same time had no dominant and varying vibrational features in the range from 1000 Hz down to 20 Hz. This was found to be necessary as the internal cooling pipes and the crystal cages were susceptible to resonances, making the whole monochromator intermittently unstable in the presence of local vibrations. The final control system chosen for the monochromator axes was the Aerotech Npaq^®^ linear amplifier system (Aerotech Inc.) but we note here that the axes were also tested and operated with a certain degree of success using the Geo MACRO system (Delta Tau, UK) together with high-resolution Renishaw interpolators (Renishaw plc). One of major issues with the Geo MACRO system was the inability to completely supress strong-intermittent and position-dependent vibrations. A schematic layout of the Aerotech control system together with the output signal it reports is shown in Fig. 3 ▸. Importantly, this jitter performance is maintained over time and, unlike other drive systems we have tested, the drive signals from the Aerotech system have been shown to have no obvious angular dependence over the magnetic poles of the drive motor.

Due to the absence of any mechanical linkage in the drive system, the pitch resolution of the axis is entirely determined by the achievable accuracy of the control system. A large 413 mm-diameter encoder ring (Renishaw plc) is mounted on a bronze disk located inside the vacuum chamber. The spacing between encoder lines is 20 µm which corresponds to 64800 encoder lines over the full circle. Four in-vacuum optical read-heads (TONiC^TM^, Renishaw plc) are then mounted at the 12, 3, 6 and 9 o’clock positions around each disk. To increase the resolution of the encoder, the analogue signal from each read-head is further interpolated 20000:1 by the Aerotech control system. The system therefore provides a total of 1.296 × 10^9^ counts over 360° with the highest angular resolution of the Bragg axes of 4.848 nrad. The working angular range of the axes covers from −1° to 33°.

**Figure 4:**
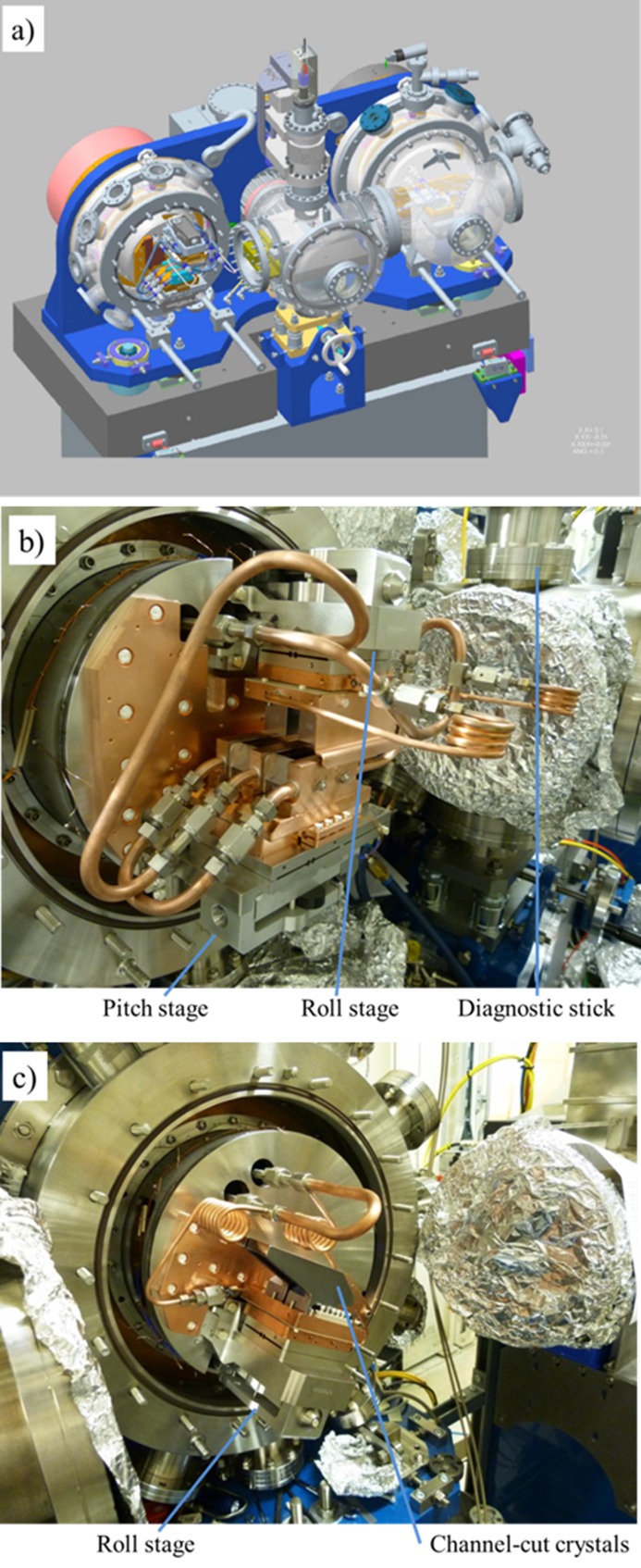
(*a*) Mechanical layout of the four-crystal monochromator. The direction of the beam is from left to right in the figure. (*b*) Photograph of the upstream axis showing the crystal cage fully assembled; an insertion diode and fluorescence screen are mounted on the diagnostic stick. (*c*) Photograph of the downstream axis showing the channel-cut crystals.

The crystal cages for the two axes of the monochromator were also designed and assembled in-house, and are shown in Fig. 4 ▸. Each crystal cage is mounted on a steel drum that is attached to the bronze disk of each axis. The liquid-nitro­gen pipes and the water manifolds used to stabilize the temperature of the assembly are brought into each axis through the shaft. The high heat load of the incident white beam requires the first-reflecting crystal to be cryogenically cooled, while the second crystal needs to be thermally isolated from the first. The third and fourth crystals are less affected by heat-load issues and therefore a channel-cut crystal has been chosen. In order to match the lattice constant of the diffracting planes, all four-crystals are directly cooled from a single liquid-nitro­gen source. The first crystals are side-clamped whilst the second and the channel-cut crystals in the second axis are base-clamped.

The use of two separate crystals for the upstream axis has made it necessary to include pitch and roll motions so we can set them parallel to each other. Thus, a pitch stage has been included in the first crystal, while a roll stage has been added to the second crystal. As was the case with the performance of the rotator axis, the performance specifications of those stages are extremely tight to ensure optimal functioning of the four-bounce monochromator. The stages carrying the first and the second crystal need to maintain the parallelism while the device is scanned through at least 2 keV, that is the extreme length of an extended absorption scan, corresponding to a maximum angular range of 10.39° (4 to 6 keV) with Si(111). At the extreme this implies a maximum drop of ±100 nrad per degree of the Bragg rotation. This was achieved using high-resolution in-vacuum piezo actuators (Piezosystem Jena GmbH) and capacitive sensors (NCDT 6500 Micro-Epsilon, UK) mounted close to the diffracting surface of the crystals. This allowed the pitch and roll stages to be operated in closed-loop, and keeps the stages at a fixed position (Duller *et al.*, 2012 ▸). The motion ranges for both pitch and roll have been kept small to allow very high resolution: the pitch stage has a full range of 4 mrad with a resolution of 90 nrad while the roll stage has a range of 5 mrad with a resolution of 112 nrad. An additional roll stage with the same characteristics has been added to the second axis, so the channel-cut crystals can be aligned in roll to the first crystal pair. The stages have been designed to use a two-flexure system. A simpler flexure hinge-based system was ruled out after the initial analysis showed that stresses in the system would compromise its performance if the piezo was acting directly on the main flexure. The whole stage assembly is mounted on a substantial T-shaped Invar plate that in the case of the pitch stage features a stiff hinge to allow the roll of the assembly to be set during installation but offering high stiffness during operation. A copper plate is bolted to the top surface of the main flexure. A heater is attached to this plate to allow it to be thermally stabilized and maintained at room temperature, minimizing the time required for the system to stabilize when the input power is varied, and helps to ensure the stability of the overall system. The cold copper plate holding the crystals is isolated from the room-temperature copper plate by three glass balls in stainless steel seats, providing thermal isolation and kinematic mounting. The crystals of the first set are placed on this copper plate. They are side-cooled with the help of three copper heat exchangers attached to the copper plate. The liquid nitro­gen flows through those heat exchangers. In the case of the second crystal set and the channel-cut crystals, the liquid nitro­gen flows directly through the cold copper base plate and the bottom of the crystals are in contact with this plate. In order to reduce the distortion in the crystal lattice when cooling, a thin foil of indium is placed in between the silicon and the copper in all cases.

The pitch adjustment in the first crystal and the roll adjustment in the second crystals and the channel cut pair, together with the two main Bragg axes, are the only mechanical adjustments in the monochromator. In order to access the wide range of angles that the I20 monochromator will operate at (−1° to 33°), the beam diffracted from the first crystal is allowed to walk across the second and third crystals. To keep the size of the second crystal relatively small and avoid problems with deformation due to gravitational sag, a relatively small gap of 8 mm between the first and second crystals has been chosen. The same gap was chosen for the channel-cut crystals of the second axis.

## 3. Alignment of the Bragg axes   

As mentioned in the section above, the number of mechanical adjustments in the monochromator was kept to a minimum in order to maintain the stability of the device. Moreover, the overall range of the few adjustments that are used in the crystal cages is very limited, 4 mrad and 5 mrad for the pitch and the roll stages, respectively, in the first crystal cage, and 5 mrad for the roll of the crystal cage in the second axis. Consequently, a critical stage of the commissioning of the four-bounce monochromator is the accurate positioning and alignment of the Bragg axes with respect of the incoming beam, both in roll and yaw. This alignment has been performed following a series of steps using a laser tracker (Leica Absolute Tracker AT901).

As a first step, a pair of reflectors was mounted on the front and back faces of the upstream Bragg axis. By rotating them, it has been possible to accurately generate the coordinates of the centre of rotation at two different points in space. These coordinates have then been used to build up a geometric model of the centreline of the axis. Using this model, it has been possible to manually adjust the whole monochromator until the axis is aligned in roll and yaw with the incoming beam. The height of the whole monochromator has then been adjusted, until the centre of rotation of the upstream axis is located on the centreline of the nominal beam position.

The next step is to adjust the downstream axis until it becomes parallel to the upstream axis, both in roll and yaw. It is possible to align the downstream axis horizontally with respect to the upstream axis because a pair of vertically reflecting mirrors is placed upstream of the monochromator to maintain the horizontal trajectory of the incoming beam. The parallelism between the two axes was carefully checked several times to improve the accuracy of the measurements. This process ensured that the roll and yaw misalignments between the two axes were kept below 60 µrad and 170 µrad, respectively. These misalignments are within the tolerances calculated theoretically (Sutter *et al.*, 2008 ▸).

Finally, the laser tracker and a small reflector were used to place the reflecting surface of the first crystal at the centre of rotation of the upstream axis and the reflecting surface of the fourth crystal at the centre of rotation of the downstream axis. The pitch and roll of the crystals were also checked and corrected using a high-precision spirit level. The miscut of the Si(111) crystals was taken into consideration when mounting the first and second crystals to the stages, so that the limited range provided by the piezo actuators was enough for the diffracting planes of the crystals to be aligned with the incoming X-ray beam. The alignment between the first and second crystals was confirmed and, if necessary, re-adjusted by passing a laser beam through the crystal surfaces and projecting the spot several metres from the monochromator.

## 4. Commissioning of the drive system   

The drive system of the Bragg axes required a considerable amount of testing and optimization due to its unique design and the use of high-precision encoder decoding. Three aspects of the performance of the axes were looked at in great detail: (i) the stability of the axes at each commanded position, which is critical to maintain synchronization of the axes over a long period of time; (ii) the linearity of long- and short-range motions; and (iii) the repeatability of step moves. Tests were performed by synchronously acquiring the positional outputs from the drive controller and external measurements from either an autocollimator (Elcomat 3000, Moeller-Wedel) or a capacitive sensor (Micro-Epsilon NCDT 6500). We note that the capacitive sensor with a linear resolution of 1.5 nm, mounted tangentially to the axis of rotation and 200 mm from the centre of rotation, provided a maximum angular resolution of 7.5 nrad.

During the tests, it quickly became evident that electrical interference and noise in the drive system and cables must be kept to a minimum to achieve the level of stability demanded by the axes. It was found that the presence of electrical noise not only enhanced the magnitude of the jitter reported by the control system but also had a tendency to drift the axis from its commanded position. To minimize electrical noise, the drive units were put in a dedicated control rack with enhanced EMC shielding and housed locally to the monochromator to keep the length of the signal cables as short as possible. This layout also allowed the signal and motor cables to be carefully segregated from the power cabling. To further ensure the static performance of the axes, a series of EMC/EMI filters, AC mains filters and isolating transformers were added to the power supply to the control drives, and shielded cables have been used for internal rack wiring. With the implementation of the above and optimizing the control-loop of the drive, the stability of the axes was improved remarkably with no measurable drift detected from the command position.

**Figure 5:**
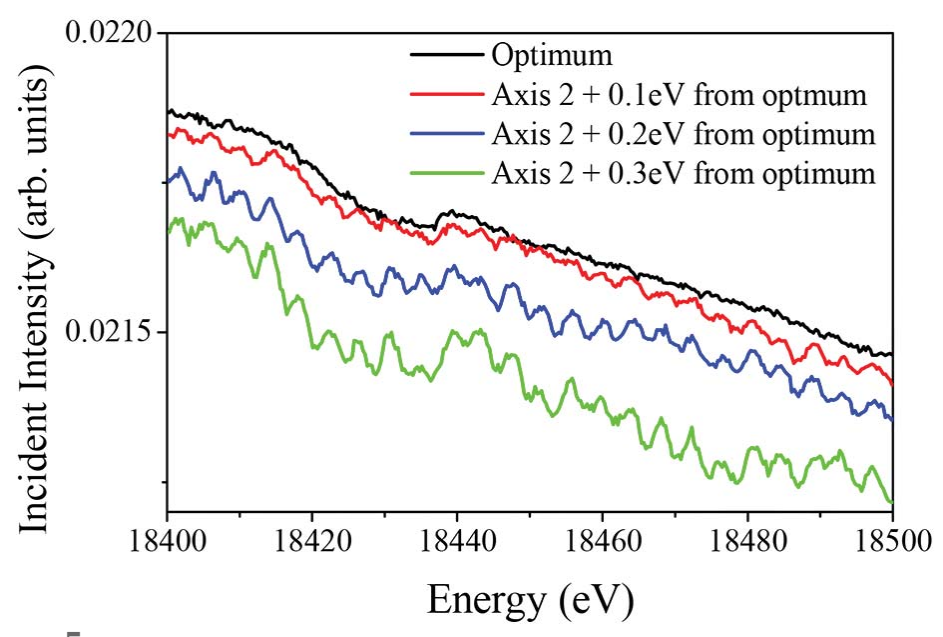
Incident intensity profile measured from 18.4 to 18.5 keV. The scans were repeated with the axes fully synchronized (black line: optimum) and with the downstream axis deliberately set away from this optimum setting. The cyclic oscillations due to the interpolation errors are only visible when the axes are not completely synchronized. An offset of 0.1 eV at this energy corresponds to about 0.6 µrad angular misalignment between the two axes.

**Figure 6:**
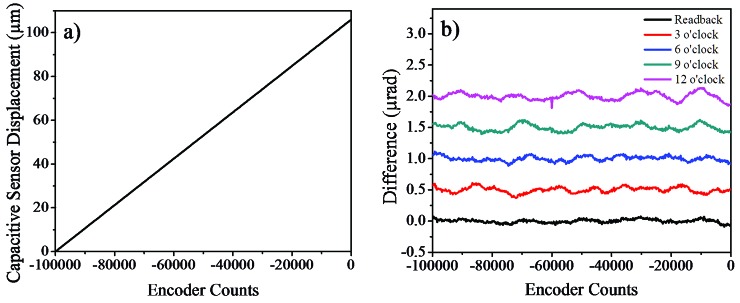
Encoder alignment measurements taken using the capacitive sensor: (*a*) an example of angular displacement measured by the capacitive sensor, showing that the long-range linearity of the axes is excellent; (*b*) the difference between the read-back values and fit to the measured data after the alignment process. The individual encoder data are shifted vertically for clarity. The presence of the interpolation (20000-line oscillations) errors is evident, with each encoder having a different amplitude and phase.

Each Bragg axis is controlled using the readouts from four encoder heads. The analogue outputs from the encoder heads are summed within the drive controller and used as positional feedback for the direct-drive motor. The principal reason for using more than one read-head was to reduce the inherent cyclic error of the high-resolution interpolator that is translated into positional fluctuations. The interpolation error is small but periodic (20000 counts, 98.48 µrad) and it is certainly significant when sub-microradian resolution is required. This is particularly true when the monochromator axes are not totally synchronized and scanned in energy as can be seen in Fig. 5 ▸. This figure clearly illustrates the effect that the interpolation errors have on the transmitted beam; cyclic oscillatory features can be seen on the incident intensity monitor when scanning the monochromator. Attempts were made to minimize these errors by monitoring the sinusoidal/Lissajous signals from each read-head and carefully aligning it, but unfortunately the ideal sinusoidal curves were never attained. However, it was found that this error could be reduced significantly if the outputs from the four read-heads were averaged and used as feedback. If the cyclic errors from the encoders have similar amplitude values, then their periodic nature opens the possibility that they will cancel each other out. This cancellation effect is most effective when the errors are out of phase, thus attempts were made to achieve this by adjusting the alignment of the read-heads. Fig. 6 ▸ shows a typical capacitive sensor scan after the read-head alignment. The alignment process took several attempts and was repeated over the full rotation range to reduce the cyclic errors to ±0.1 µrad from each encoder head.

An additional benefit of using multiple read-heads on the same encoder ring is the reduction of long-range following errors introduced by graduation errors of the encoder ring. No encoder rings are ever perfectly circular or concentric due to manufacturing and installation inaccuracies; consequently each encoder ring has a unique graduation pattern which varies along its circumference. The exact profile of graduations is dependent on the amount of tension used to secure the encoder ring but a graduation accuracy of approximately ±2 µrad over the entire range is quoted by the manufacturer of the encoder rings used (http://www.renishaw.com). The total radial run-out of the encoder ring installed on the bronze disk was approximately ±2.5 µm. It is clear that if the spacing of the encoder lines is non-uniform, then it will introduce a discrepancy between the commanded value and the actual axis position. This will cause the Bragg axes to progressively lose their synchronization as they are rotated together from a calibrated position. This issue can be overcome by generating a look-up table of axis positions as a function of energy as the graduation error is repeatable, but averaging the outputs from four spatially separated read-heads will also significantly reduce the effect of this error.

**Figure 7:**
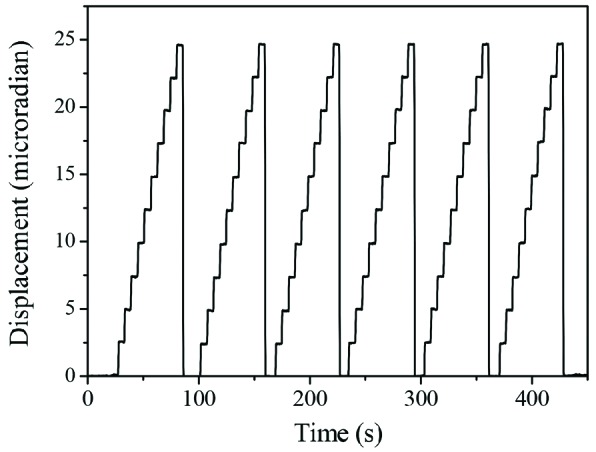
Step scans measured using the capacitive sensor. The same scans were repeated six times to demonstrate the repeatability of the axis. These data have been recorded using the Geo MACRO drive with Renishaw 2000-line interpolators.

A series of step scans measured using the capacitive sensor is shown in Fig. 7 ▸. The capacitive sensor was mounted tangentially to the rotation axis to allow angular displacement measurements. The measurements were taken by commanding the axis to move from 0 to 24.2 µrad with a step size of 2.424 µrad (50 interpolation lines with 2000-line decoding), which corresponds to approximately 0.12 eV at 10 keV with a Si(111) crystal cut, and a step time of 5 s. It can be seen in Fig. 7 ▸ that the axis is moving accurately to the intended positions without any significant time-delay, backlash or overshoot, and that it maintains excellent positional stability between successive steps. The largest positional variation observed from six repeated scans (total 60 steps) was only ±90 nrad, which is remarkably consistent and demonstrates that the mechanical repeatability of the axis is better than sub-microradian under the normal mode of operation.

## 5. Experimental data   

**Figure 8:**
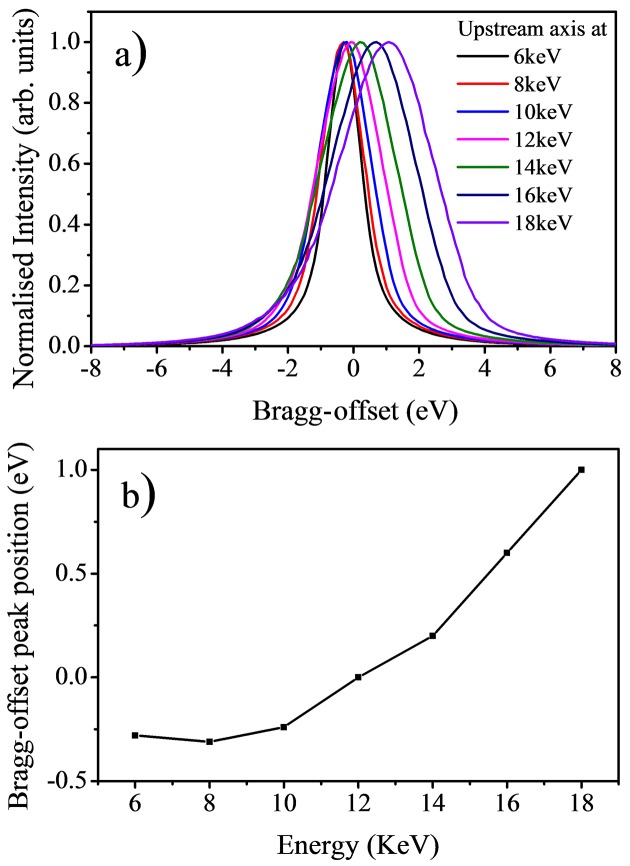
(*a*) Measured Bragg-offset scans at 6, 8, 10, 12, 14, 16 and 18 keV with the Bragg-offset value set to zero at 12 keV. (*b*) The maximum intensity position of Bragg-offset scans as a function of energy.

The long-range linearity of the Bragg axes can be characterized by looking at how the optimum offset value between the axes changes with energy. Fig. 8 ▸ shows the intensity throughput at several energy values between 6 and 18 keV. The intensity is measured by fixing the upstream axis at a given angular value and rotating the downstream axis from −8 to +8 eV around the same angular value. This type of diagnostic scan is known as a Bragg-offset scan and is used to find the optimum synchronized position of the axes by identifying the angular value at which the intensity transmitted by the two axes is at its maximum value. In the case of Fig. 8 ▸, the offset between the Bragg axes (Bragg-offset) was set to zero at 12 keV, and Bragg-offset scans were performed at several energy values from 6 (19.24°) to 18 keV (6.31°). If the two Bragg axes were totally linear, the offset value between the axes will remain at zero for each energy position. Although no additional external feedback (intensity or positional) was used to preserve the synchronization of the axes, we see that the optimum Bragg-offset values are only varying from −0.3 eV at 6 keV to +1.0 eV at 18 keV. In fact, this change is sufficiently small that we can just scan the axes from 6 to 18 keV without completely losing the flux throughput with the optimum throughput position found at 12 keV. This shows that the two Bragg axes are remarkably linear over this wide energy range and the encoder system used to maintain the axes synchronized in energy is operating robustly.

Despite the remarkable linearity of the Bragg axes, the offset between the two axes is adjusted at every energy point of an absorption scan when the monochromator is operating. This correction accounts for any slow drift in the incident beam vertical angle due to sub-microradian variations in the beam orbit of the synchrotron storage ring and/or pitch angle of the upstream optics. The fine optimization of the Bragg­offset is carried out by measuring the offset value at the initial and at the final energy of the absorption scan. The Bragg-offset value at every data point is then linearly interpolated from the start and end values to better maintain the synchronization of the Bragg axes.

**Figure 9:**
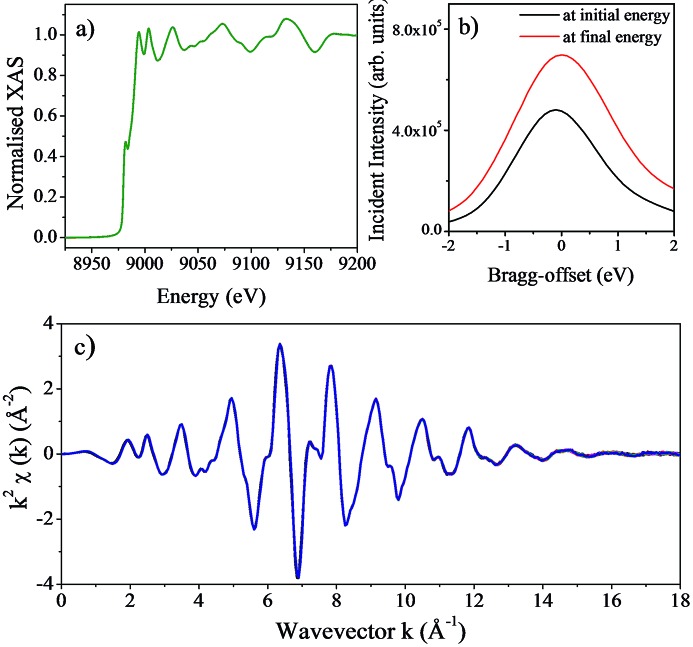
(*a*) X-ray absorption spectra of a Cu foil; 25 consecutive scans are shown. The spectra have been normalized by setting the edge step to a value of 1. No alignment of the spectra has been performed. The XANES edge features are clearly visible demonstrating the excellent energy resolution of the I20 four-bounce monochromator. (*b*) Bragg-offset scans taken at the initial (black line) and final (red line) energies for the first scan in the series. (*c*) Extracted EXAFS signal of the spectra shown in (*a*), showing the excellent reproducibility of the 25 scans collected.

To acquire a good quality X-ray absorption spectrum, the beamline optics must deliver high-purity monochromatic X-rays that are well calibrated in energy. Another necessary characteristic is the reproducibility of scans as typically X-ray absorption spectra are obtained by averaging several repetitive scans to improve the signal-to-noise ratio and statistical accuracy of the measurements. Fig. 9 ▸ shows a set of XAS measurements taken from a copper foil during the commissioning of the monochromator. These measurements were taken using the Si(111) crystals with the sample at the nominal sample position of the scanning branch of I20, located at 30.95 m from the monochromator. Ion chambers were used to measure the incident intensity (*I*_0_) and the intensity transmitted (*I*_t_) by the foil. The monochromator was calibrated at the Cu *K*-edge energy (8979 eV) and the offset between the two axes was set to zero at this energy prior to recording these measurements. To verify the stability and reproducibility of the scans, the same scan was repeated over a time span of 14 h, with each spectrum taking approximately 34 min. The pitch of the first crystal pair was set to be constant for the duration of the measurements but the offset between the two Bragg axes (Bragg-offset) was adjusted. For the copper foil scans presented here, the angular range covered from 13.09° (8729 eV) to 11.07° (10300 eV) and the difference in the Bragg-offset value at the start and end of each scan was only 0.11 eV.

**Figure 10:**
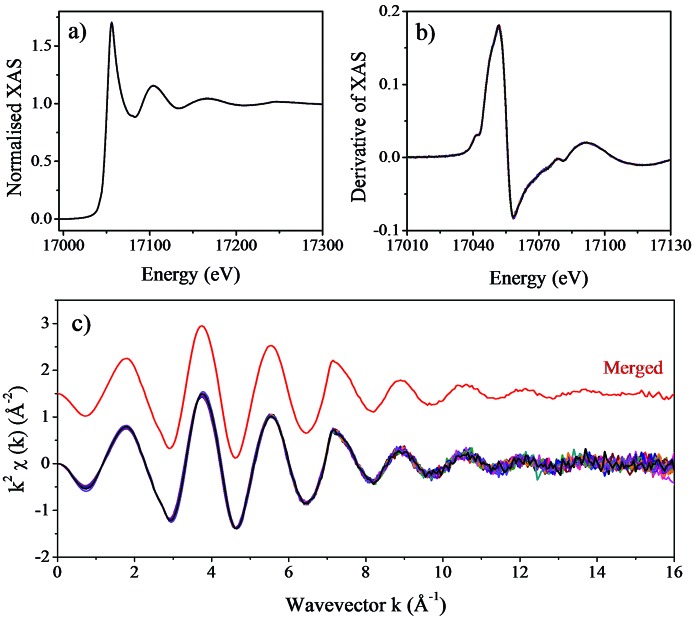
(*a*) X-ray absorption spectra of a 1 m*M* yttrium chloride aqueous solution collected in fluorescence detection mode. Fifteen consecutive scans taken from this sample are shown. (*b*) First derivative of the 15 scans collected. The excellent energy stability of the monochromator can be seen from the peak position of the first derivative, unchanged over 10 h. (*c*) Extracted EXAFS signal of the spectra shown in (*a*), including the merged of the 15 spectra collected (red line).

XAS measurements taken from a dilute yttrium chloride (1 m*M* YCl_3_) aqueous solution are shown in Fig. 10 ▸. These measurements were taken in fluorescence detection mode using a multi-element solid-state germanium detector (Canberra). The sample was placed in a quartz capillary and the measurements were performed at room temperature. The monochromator was calibrated at the Y *K*-edge (17038 eV) and the offset between the two axes was set to zero at this energy prior to taking these measurements. In order to improve the statistical accuracy of the EXAFS signals at high-*k* values, 15 consecutive scans were taken over a time span of 10 h with each scan taking approximately 40 min to collect. As with the previous copper foil data, the synchronization of the Bragg axes was maintained using the Bragg-offset optimization routine for the duration of the measurements. We can see from Fig. 10 ▸ that the consistency of the EXAFS spectra is excellent and we can safely merge the measurements taken with this monochromator without the need of aligning the spectra to obtain a reliable spectrum. A further verification of the energy stability of the monochromator is provided by the reproducibility of the XANES spectra, showing that the edge position is repeated to better than ±0.10 eV over a long period of time.

## 6. Conclusions   

We have successfully overseen the principal challenge of developing and commissioning a four-bounce monochromator that can maintain the flux throughput while scanning a wide energy range. The excellent energy stability and reproducibility needed to perform absorption measurements have been achieved, differentiating the I20 monochromator from other four-crystal devices which require regular or real-time readjustment routines to maintain the two axes synchronization (Krumrey, 1998 ▸; Trela *et al.*, 1988 ▸; Heald *et al.*, 1986 ▸; Heald, 1988 ▸; Kraft *et al.*, 1996 ▸; Tolentino & Rodrigues, 1992 ▸). The exceptional mechanical performance of the axes also makes this design suitable for any other application that demands precise sub-microradian positioning, high resolution and stability. As a matter of fact, a number of double-bounce monochromators that utilize the design principles of these Bragg axes have already been commissioned and they are now successfully operating at Diamond Light Source. The I20 monochromator was the first monochromator that has been designed, developed and commissioned in-house at Diamond Light Source.
